# Rethinking Osteoporosis Drugs: Can We Simultaneously Address Sarcopenia?

**DOI:** 10.3390/ijms26146924

**Published:** 2025-07-18

**Authors:** Zoran Gavrilov, Jasna Lojk

**Affiliations:** 1 Lab for Retinal Gene Therapy, Department Ophthalmology, University Hospital Zurich, University of Zurich, Wagistrasse 14, 8952 Schlieren, Switzerland; zoran.gavrilov@uzh.ch; 2 Department of Clinical Biochemistry, Faculty of Pharmacy, University of Ljubljana, Aškerčeva cesta 7, 1000 Ljubljana, Slovenia; 3 Clinical Institute for Clinical Chemistry and Biochemistry, University Medical Centre Ljubljana, Njegoševa cesta 4, 1000 Ljubljana, Slovenia

**Keywords:** osteoporosis, sarcopenia, osteosarcopenia, pharmacological treatment

## Abstract

Osteoporosis and sarcopenia are two aspects of the geriatric syndrome that frequently occur together and affect one another in a condition referred to as osteosarcopenia. Preventive and treatment options for osteosarcopenia exist but are mainly focused on the treatment of osteoporosis, as there is still no FDA-approved treatment for sarcopenia. Drugs for osteoporosis include antiresorptive and anabolic drugs and hormonal replacement therapies and are prescribed based on age, BMD and other patient characteristics, which, however, do not include the possible co-existence of sarcopenia. As several studies and clinical trials have shown that the pharmacological treatment of osteoporosis can also affect muscle tissue, in either a positive or negative manner, sarcopenia should be another factor affecting the choice of treatment, especially when facing equal treatment options for osteoporosis. The aim of this review was to summarize our current knowledge on the effects of FDA-approved drugs for the treatment of osteoporosis on muscle quality, mass and function. A better understanding of the effects that certain drugs have on muscle tissue might in the future help us to simultaneously at least partially also address the wasting of muscle tissue and avoid further pharmacologically induced decline.

## 1. Introduction

Osteoporosis and sarcopenia are age-related musculoskeletal diseases that affect a high number of people older than 65 years as an important part of geriatric syndrome. As the two diseases frequently occur simultaneously, the term osteosarcopenia was proposed in 2009 [1] and describes a patient suffering a combination of low bone mineral density (BMD) (osteopenia/osteoporosis) and low muscle mass, strength and/or functional capacity (sarcopenia) [2]. Osteosarcopenia leads to frailty, the loss of independence, poor quality of life, an increased probability of falls and more frequent osteoporotic bone fractures, which can result in lower life expectancy or even death. It is estimated that osteosarcopenia affects 1.5–65.7% of community-dwelling older people, and it is diagnosed at even higher rates in people with low-energy trauma fractures (7.1–96.3%) [2,3]. In 2010, it was estimated that 5.5 million men and 20 million women were affected by osteoporosis in Europe alone, which resulted in approximately 3.5 million fragility fractures (including 610,000 hip fractures) and cost over EUR 37 billion [4]. Moreover, osteosarcopenia and limited mobility also cause social isolation, accelerate frailty, worsen chronic health issues (e.g., hypertension, cardiovascular diseases) and diabetes and cause a decline in mental state (depression, neurodegenerative diseases) [5,6]. As such, the disease represents a significant socio-economic and health care problem, and with the increasing aged and obese population, in the next decades, the costs are estimated to increase significantly [4]. A unified preventive strategy for maintaining mobility, cognitive function and independence could not only help delay the onset of osteosarcopenia but also reduce other symptoms of geriatric syndrome and enable elderly people to maintain a higher quality of life for longer.

Preventive and treatment options for osteosarcopenia exist; however, they are mainly focused on the treatment of osteoporosis. While preventive recommendations (regular physical activity, optimal nutrition intake, protein-rich diet and avoidance of alcohol and smoking) merely delay the onset of osteosarcopenia and slow down its progression, pharmaceutical-based treatments are only prescribed to address the deterioration of bone tissue. FDA-approved drugs for osteoporosis include antiresorptive, anabolic and hormonal replacement therapies [7]; however, despite increasing research, there is no FDA-approved treatment for sarcopenia yet. This is partially due to the fact that sarcopenia was recognized as an independent condition with an International Classification of Disease-10 code ICD-10-CM (M62.84) only in 2016 [8], allowing physicians to formally include sarcopenia in the list of diagnoses that can be used and funded. However, despite revised recommendations on diagnosis and treatment from the European Working Group on Sarcopenia in Older People (EWGSOP2) in 2019 [9], integrating sarcopenia into clinical practice remains challenging. Many physicians in clinical practice are not aware or do not have the resources necessary for the screening and diagnosis of sarcopenia, muscle strength and quality are technically difficult to measure and interpret accurately [10,11], and no diagnostic panel of markers has been approved so far [12]. Many patients thus go undiagnosed, hiding the true prevalence and impact of osteosarcopenia.

Fortunately, more research attention has been given to these diseases in the last decade, and the new information on the genetic and physiological mechanisms of disease development and progression represents an invaluable source of possible novel treatments and preventive measures. Like bone and muscle tissues, osteoporosis and sarcopenia are tightly connected. They share many risk factors, signaling molecules, biological processes and pathways as well as similar genetic predispositions, originating from the common mesenchymal origin and early cell differentiation events. This gives us a unique opportunity to find common mechanisms and determinants that will allow us to address both diseases at the same time, possibly treating them with the same drug. Moreover, the interconnection of both tissues will hopefully allow for a positive feedback loop. Improvement in the quality of one tissue may promote improvement in the other and vice versa, boosting the treatment efficacy.

The aim of this review is to summarize our current knowledge on the effects of FDA-approved drugs for the pharmacological treatment of osteoporosis on muscle quality and function. When faced with equal treatment options, a better understanding of the effects certain drugs have on muscle might influence treatment decisions for patients with osteosarcopenia in the future. In this way, sarcopenia could be at least partially addressed with the same treatment, as FDA-approved drugs for sarcopenia are not available yet.

## 2. The Bone–Muscle System

**Figure 1:**
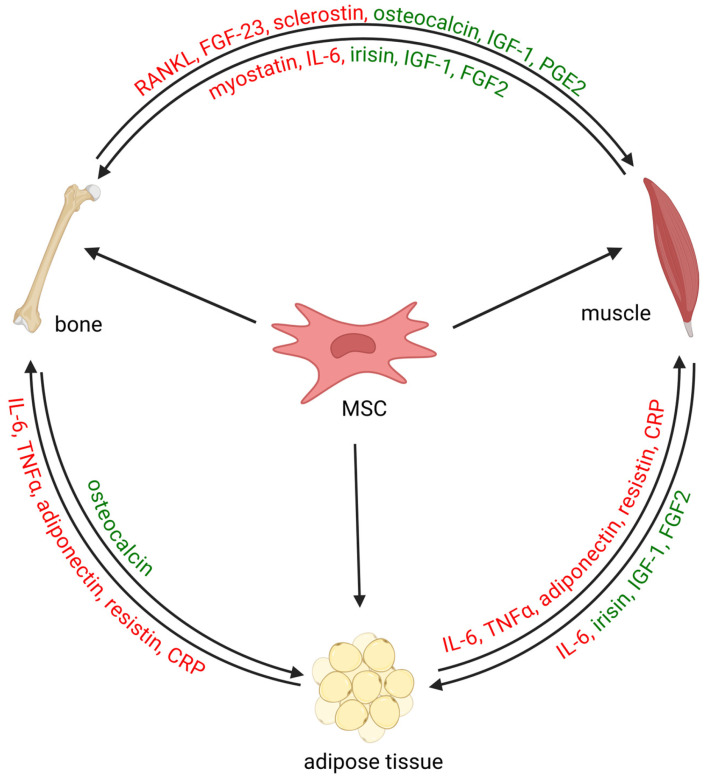
Schematic representation of cross-communication between bone, muscle and adipose tissues. Bone, muscle and adipose tissues, all originating from mesenchymal stem cells (MSCs), interact via secreted factors that exert anabolic (green) or catabolic (red) effects on each other. The figure illustrates how myokines (e.g., myostatin, interleukin 6 (IL-6), irisin), osteokines (e.g., osteocalcin, IGF-1, RANKL) and adipokines (e.g., TNFα (tumor necrosis factor α), adiponectin, resistin) mediate reciprocal anabolic or catabolic effects. The dysregulation of this signaling network plays a central role in aging and metabolic disease progression.

Bone and muscle tissues are recognized as interacting and tightly connected tissues, not only due to their proximity and mechanical stimulations but also through genetic, paracrine and endocrine signals that coordinate development, regeneration and response to mechanical forces [13]. This connection originates from a common mesenchymal progenitor cell type during embryogenesis and consequently shared patterns of gene expression and signaling pathways [14], such as growth hormone/insulin-like growth factor-1 (GH/IGF-1), steroid sex hormones and Wnt (wingless-type MMTV integration site family)/Hippo signaling pathways. This endocrine communication between the bone and muscle tissues is regulated through the secretion of myokines and osteokines, which can have anabolic or catabolic effects on bone and muscle tissue turnover as well as the whole body (Figure 1) [15]. For example, muscle-secreted myostatin has negative effects on both muscle mass and bone formation through the inhibition of osteoblast differentiation [16,17]. On the other hand, muscle exercise induces the secretion of IGF-1 [18], basic fibroblast growth factor (FGF-2) [19] and irisin [20], has positive osteogenic effects and can decrease adiposity through an increase in osteoblast survival, proliferation and differentiation. Bone tissue also acts as endocrine organ, and cells secrete osteokines such as osteocalcin, IGF-1 and prostaglandin E2 (PEG2), which positively affect muscle quality and function, while nuclear factor-kappa B receptor activator ligand (RANKL; TNFSF11), FGF-23 and sclerostin (SOST) inhibit myogenic differentiation [21].

A consequence of low physical activity and dysregulated tissue metabolism is also the accumulation of adipose tissue, which is recognized as a common aging process and is also referred to as osteosarcopenic obesity. Bone marrow adipogenesis is accompanied by reduced BMD, reduced bone formation and increased fracture risk [22,23], while fat infiltration in skeletal muscle (myosteatosis) is associated with the decreased sensitivity of muscle cells to insulin, inflammation and decreased tissue quality, strength and endurance [24,25]. Increased adipogenesis is a consequence of the dysregulation of mesenchymal stem cells (MSCs), the common precursor cells for osteoblasts, myoblasts and adipocytes, which switch towards adipogenesis under aging conditions [26], such as a reduction in estrogen or androgen levels, lower physical activity and lower nutritional intake. Lipid accumulation is associated with the release of toxic fatty acids [27], inflammation [28] and lower tissue regeneration abilities, as the number of stem cells decreases or MSCs do not activate from their quiescent state due to unfavorable microenvironmental conditions in tissues [29].

Adipose tissue is also a source of proinflammatory signaling factors and plays an important part in age-related chronic (low-grade) systemic inflammation, also referred to as “inflammaging”. This chronic inflammatory state has been linked to dysfunction in tissue repair and renewal, increased bone and muscle loss and changes in energy metabolism and senescence, which lead to accelerated aging. Osteosarcopenic patients have higher concentrations of adiponectin, brain-derived neurotrophic factor (BDNF), interferons, tumor necrosis factor α (TNF-α) and several interleukins (IL-2, -4, -5 and -10), especially IL-6 and IL-8, compared to healthy age-matched controls [30]. In bone tissue, low inflammatory concentrations of IL-6, TNF-α and IL-1β are associated with the promotion of osteoclast differentiation, migration and activity as well as the inhibition of osteoblast differentiation, which is mediated through the increase in RANKL secretion and changes in several signaling pathways [31,32]. This increased bone resorption leads to the progressive loss of bone mass and BMD and increased bone fragility. In muscle tissue, chronic low inflammation is associated with muscle atrophy, the loss of muscle strength and insulin resistance but also leads to senescence. Cell senescence is characterized by cell cycle arrest in satellite cells, changes in the metabolic rate, increased oxidative stress and DNA damage and changes in cell signaling and DNA expression, which lead to the additional secretion of proinflammatory cytokines, potentially causing the senescence-associated secretory phenotype (SASP) [33]. Chronic inflammation is thus an important mechanism that disrupts musculoskeletal homeostasis and regeneration, promoting the emergence of osteosarcopenia. Unfortunately, chronic inflammation is not currently directly addressed in clinical treatment and prevention guidelines for osteoporosis and sarcopenia [34,35,36,37], which is another important area for future improvement in patient care.

Mechanical forces in the form of mechanical loading, weight bearing and physical impacts, which are an important part of bone–muscle development and turnover, are another important connecting factor. Starting from embryonal development, when mechanical forces from developing muscle tissue stimulate periosteal bone growth and are essential for adequate bone density and bone geometry [38], mechanical loading and unloading, determined by muscle activity and the strength and type of movement, are one of the main driving factors of bone turnover and the maintenance of BMD [39]. Mechanical stimuli increase the Wnt and Hippo signaling activity in osteocytes [39], which alters the processes of bone catabolism and anabolism to adjust bone strength. Similarly, mechanical loading also increases muscle size and strength through increased cell growth, cell differentiation and matrix remodeling [40]. Thus, when loading and physical activity are decreased, this affects both tissues.

Bone and muscle tissues thus share many genes, pathways and mechanisms, which is also reflected in shared risk factors and preventive measures for osteoporosis and sarcopenia, providing the basis for possible novel approaches to treat both diseases simultaneously. Unfortunately, through this mutual communication, adiposity, sarcopenia and osteoporosis exacerbate each other in a vicious cycle of metabolic and inflammatory effects that stimulates the catabolism of bone and muscle tissues and disease progression [41]. Mechanisms that delay or reverse these changes are thus at the core of any preventive measures and could also represent a supportive therapy to enhance the effects of pharmacological treatments.

## 3. Preventive Measures and Non-Pharmacological Approaches for Osteosarcopenia

Physical exercise is one of the most effective ways to prevent or delay osteosarcopenia, reduce tissue adiposity, maintain the strength required to perform daily activities and reduce the risk of falling. Numerous studies have been conducted over the last few decades using different exercise equipment and training regimens, most of them showing significant improvements in tissue quality and general health independent of age. In muscle tissue, aerobic exercises stimulate mitochondrial biogenesis and activity [42,43] and changes in the expression of important protein markers [44] and reduce cell apoptosis. This results in increased aerobic capacity and a minor increase in muscle strength, mass and volume [44,45,46]. On the other hand, resistance exercises stimulate muscle hypertrophy and increase muscle strength and function mainly through an increase in muscle protein synthesis [47,48]. Even low-intensity activities like daily walking have been shown to decrease the risk of falling and hip fracture in elderly people [49,50]. Resistance-based physical activity is also the only strongly recommended preventive and treatment approach for sarcopenia in the recent guidelines of the International Conference on Frailty and Sarcopenia Research (ICFSR) task force [35].

While muscle growth requires primary resistance and aerobic training, bone tissue growth requires resistance loading and physical impacts, which induce bone micro-deformations [51]. For example, low-resistance exercises with no impacts, such as cycling or swimming, despite inducing a considerable gain in muscle mass, were associated with normal-to-low BMD [51,52]. Similar results were also obtained with whole-body vibration therapy, which was proposed as a safe and low-demand alternative to physical exercise, especially for people who cannot exercise effectively [53]. The effects of exercise are also strongly dependent on age. While in younger people strength/resistance and weight-bearing exercises increase the BMD and bone mass to the optimal level for each individual and stabilize them, the increase in elderly people is usually smaller or absent, and the exercises seem to be more efficient in preserving the BMD gained during youth/earlier life than in reversing age-related BMD loss [54,55]. This could explain the inconsistences obtained in several clinical trials of different exercise regimens but also points to the importance of adequate physical activity during youth and adult life.

Unfortunately, despite its positive effect on its own, physical exercise is rarely applied as a supplementary treatment alongside prescription drugs for osteoporosis. This is partially due to the fact that most patients with osteoporosis are diagnosed only upon low-energy bone fractures, after which exercise might not be possible any more, but osteoporosis also has a high rate of discontinued treatments [56]. Only a few studies so far have addressed the effects of combined drug and exercise treatment, but a small or no synergistic effect on BMD was detected. A recent meta-analysis combined the results of nine studies addressing the combined effects of different antiresorptive drugs (hormone replacement therapy [HRT], isoflavones, bisphosphonates) and different types of exercise and showed that the combination of antiresorptive agents and exercise generated additive effects on lumbar spine BMD but only showed a non-significant positive effect on femoral neck BMD. Due to the high heterogeneity between studies, the authors were unable to determine the most efficient drug treatment but showed that impact exercise interventions had a more synergistic effect with antiresorptive drugs compared to resistance training [57].

Another modifiable risk factor for osteosarcopenia is the lack of adequate nutrition, which is common in elderly people [58]. The main nutrients required for bone and muscle health are vitamins (especially vitamin D3 and vitamin K), inorganic minerals (including calcium and phosphates) and macronutrients, such as proteins and fatty acids, all of are required in sufficient amounts for optimal tissue regeneration and growth [59,60]. The European Food Safety Authority (EFSA) recommends a minimal 25(OH)D serum concentration of 50 nmol/L for all groups [61], which can be achieved with 600–800 IU/day, but the dose should be increased in conditions of low sun exposure or older age, when the absorption, kidney hydroxylation and skin production of 25(OH)D are lowered [62]. Sufficient 25(OH)D and protein supplementation was also observed to lower the chronic low-grade inflammation processes in sarcopenic individuals by reducing the secretion of inflammatory cytokines and increasing the production of IGF-1 [59]. However, especially regarding bone health, in order to increase BMD and reduce fracture risk, the supplementation of calcium, the main component of the inorganic part of bone tissue, is also required at a recommended minimum daily dose of 1200 mg [63]. Calcium also plays an important role in muscle contraction and could thus have a positive role in mitigating sarcopenia, as suggested by a few observational studies performed so far [64], but no clinical/therapeutic data is available.

Similarly, a lower protein intake was associated with lower BMD, a higher risk of hip fracture and worse outcomes after hip fracture in several studies [65,66]. Sufficient protein intake provides the amino acids used in building and maintaining bone and muscle tissue and increases the secretion of IGF-1, which in turn promotes bone growth and increases calcium absorption [67]. International expert groups recommend a daily protein intake of 1–1.2 g/kg body weight per day for people under the age of 65 to maintain and regain bone and muscle mass and function, and the intake should be increased to 1.2–1.5 g/kg body weight per day in physically active or older people, especially those with acute or chronic diseases [68,69]. Supplementation, of course, is not a substitute for a healthy diet and lifestyle, as it does not mitigate the negative effects of the excessive consumption of coffee and alcohol and smoking, which are also risk factors for osteosarcopenia.

Studies addressing the effects of nutrition suggest that for the best effects, all nutrients must be present in optimal amounts simultaneously. Only partial or no supplementation (in most studies, proteins are not supplemented or are increased through regular diet) could explain the inconsistencies and low reproducibility of the results. Then again, supplementation alone, without exercise, has little to no effect on muscle mass and strength [70]. Clinical trials have also suggested that supplementation can have a positive additive effect alongside drug treatment in both muscle and bone in bisphosphonate-treated osteoporotic patients [71,72,73]. For better treatment efficacy, patients should thus be encouraged to supplement crucial nutrients (vitamins, proteins, minerals) and exercise as much as their health status allows.

## 4. Therapeutic Treatment Approaches

While non-pharmaceutical treatment approaches help, they are not robust enough to prevent or even reverse quickly progressing osteosarcopenia, fueled by other concomitant diseases, or already advanced sarcopenia and frailty. In such cases, pharmaceutical treatment approaches are needed; however, so far, only drugs addressing osteoporosis are being used in routine clinical practice [34,36,37]. Based on their primary mode of action, osteoporosis drugs are classified as antiresorptive drugs (hormonal therapy, selective estrogen receptor modulators [SERMs], bisphosphonates and Denosumab), which inhibit the process of bone resorption mediated by osteoclasts, and anabolic drugs (teriparatide, abaloparatide and romosozumab), which stimulate novel bone formation by promoting osteoblast function. Treatment is selected based on the severity of osteopenia, fracture risk or already-present fractures, tolerability, previous medication, patient preferences, age, gender and other medical issues, including the ability of the patient to swallow pills or adhere to the prescribed treatment [74]. The current guidelines do not address the simultaneous presence of sarcopenia, nor is sarcopenia a factor in the Fracture Risk Assessment Tool (FRAX) [75], although it presents a significant risk for falls and thus fractures. Moreover, certain osteoporosis drugs have been shown to also affect muscle tissue, either in a positive or negative way. A drug-induced decrease in muscle quality in an osteosarcopenic patient, although protecting bone tissue, could increase the risk of falls and have an overall negative effect on the patient.

Sarcopenia was only recently recognized as a disease, which allowed for more targeted research and diagnostics. Despite intensive research and several excellent candidate drugs with promising results in clinical trials, no sarcopenia-targeting drugs have been FDA- or EMA-approved so far. Thus, taking advantage of the potential positive effects of certain osteoporosis drugs on muscle tissue might help us bridge the current gap in sarcopenia treatment by also considering the presence of sarcopenia as one of the factors affecting the selection of the most optimal drug treatment for a certain patient, especially where multiple equivalent options are available. As a relatively novel and “off-label” approach, unfortunately, not many clinical studies have addressed the effects of different osteoporosis treatments on muscle tissue, and for most drugs, there is not enough information available to make any recommendations for changes in patient treatment. As such, the following chapters addressing the effect of currently FDA-approved drugs for the treatment of osteoporosis on muscle tissue should be considered as a conceptual framework for future research and consideration. In the future, more prospective, robust, gender-specific and methodologically strong clinical studies should be performed with muscle function and quality as one of the primary outcomes before any definite conclusions, clinical recommendations and guidelines can be made. Thus, for now, any treatment decisions should first and foremost be based on currently valid clinical guidelines. Only when facing equal clinical decisions, the presence or absence of sarcopenia could be considered when deciding on any patient treatment.

### 4.1. Denosumab

Denosumab is a drug based on a human monoclonal antibody against RANKL [76,77] that was FDA-approved for the treatment of osteoporosis in postmenopausal women with a high risk of fractures [78]. RANKL is part of the RANK/RANKL/osteoprotegerin (OPG) signaling pathway, which activates the NF-κB transcription factor and in bone tissue plays a crucial part in osteoclast differentiation, activation and resorption functions. RANKL expression increases in postmenopausal women due to the reduction in estrogen levels and is the driving force behind postmenopausal osteoporosis [79]. By binding RANKL, Denosumab blocks the activation of the RANK signaling pathway and reduces osteoclastogenesis and bone resorption. Subcutaneous administration leads to the rapid onset of osteoclast inhibition, which leads to a significant increase in BMD at all observed sites (femoral neck, lumbar spine, hip, trochanter and total body) and a reduction in hip, vertebral and non-vertebral fractures, with almost no adverse effects [80,81]. In the phase 3 clinical trial FREEDOM (Fracture Reduction Evaluation of Denosumab in Osteoporosis every 6 Months) [80], a randomized, placebo-controlled trial in 7868 women with postmenopausal osteoporosis, Denosumab treatment showed a 68% reduction in the incidence of new vertebral fractures, a 40% reduction in hip fractures and a 20% reduction in other non-vertebrate fractures. It also significantly improved bone turnover markers. These positive effects, including a continuous increase in BMD without a plateau, could also be observed after 7 years of treatment, indicating that Denosumab can be used as a long-term drug [82].

RANK is also expressed in skeletal muscle but controls different intracellular processes. The direct role of RANKL in muscle wasting was shown with transgenic mice overexpressing human RANKL, which accurately recapitulated the profile of sarcopenia: decreased muscle metabolism, decreased muscle mass, insulin resistance, fat infiltration and an increase in inflammation markers [83]. Only a few clinical studies so far have analyzed the effects of Denosumab on muscle tissue (Table 1). Bonnet et al. showed that in older women with postmenopausal osteoporosis, prolonged Denosumab treatment improved appendicular lean mass and handgrip strength, which was not observed in the bisphosphonate treatment group or control group [83]. Two other studies showed that Denosumab treatment improved gait speed and Four-Square Step Test (FSST) and Timed Up and Go (TUG) test performance, reduced the fear of falling and increased the confidence of participants [84,85]. On the other hand, a recent 2-year study of elderly patients in long-term care communities comparing a Denosumab treatment group with a placebo group found no significant differences in appendicular lean mass, lower-extremity lean mass, grip strength, chair stand test performance, gait speed and short physical performance battery (SPPB) in both male and female patients, although a non-statistically lower decline in chair stand-up time and grip strength was found in treated women [86]. The authors attribute this to the low physical activity of the care facility residents, but this also emphasizes the complex mechanisms and interactions between Denosumab and muscle tissue and the need for further studies, also addressing the role of physical activity and other patient characteristics in the observed outcome.

Alongside its positive effects, Denosumab is a relatively safe drug, although certain downfalls and possible concerns are present. Denosumab is not retained in bone, and its duration of effect is short and reversible once discontinued [87,88], including a higher rate of vertebral fractures. The medication thus requires continuous administration, despite the possible side effects, which are usually lessened by regular temporal discontinuations of treatment. Comparisons with other antiresorptive drugs which increase BMD but do not affect muscle tissue [83] also suggest that the potential improvement in bone and muscle tissue following Denosumab treatment occurs in parallel and independently—any improvement in muscle function is thus not a consequence of improving bone tissue or vice versa. Despite promising results, additional studies are required to confirm the observed effects of Denosumab on muscle tissue and better assess the mechanisms through which Denosumab affects muscle tissue quality and metabolism.

**Table 1:** Overview of clinical studies addressing the effects of Denosumab on bone and muscle tissue. Abbreviations: BMD—bone mineral density; LBM—lean body mass; TUG test—Timed Up and Go test; ALM—appendicular lean mass; FSST—Four-Square Step Test; SPPB—short physical performance battery.

Cohort	Study	Regime	Effects on Bone	Effects on Muscle	Ref.
135 elderly osteoporotic patients without fractures	Longitudinal, multicenter, controlled, prospective study	60 mg every 6 months for 5 years (+ Ca and vitamin D)	Increase in BMD (spine and hip) and decreased fraction risk	Improved grip strength (+4.3 kg), TUG test (1.5 s) and gait speed (0.1 m/s), which significantly worsened after discontinuation of Denosumab treatment	[88]
60 postmenopausal osteoporotic Korean women	Prospective multicenter cohort study	60 mg every 6 months for 3 years (+ Ca and vitamin D)	Increase in BMD in lumbar spine (9.7%) and hip (5.1%)	Significant increase in fat-free mass (3.6%)	[89]
18 postmenopausal osteoporotic women (mean age 65.0 ± 1.5 years)	Retrospective—GERICO	60 mg every 6 months for an average of 3 years	Increase in lumbar spine BMD (0.12 ± 0.29 g/cm^2^)	Significant increase in ALM (0.66 ± 2.2 kg) and in handgrip strength (3.22 ± 10.0 kg)	[83]
60 osteoporotic or osteopenic patients	Retrospective, propensity-score-matched cohort study	60 mg every 6 months for an average of 1.5 years (+ vitamin D)	Annual increase in femoral (+1.83%) and spinal BMD (3.30%)	Significant annual increase in grip strength (+5.14%) and in chair-rising test force (+8.20%); no change in chair-rising test time	[85]
51 community-dwelling elderly patients (≥65 yo) with history or risk of falls and/or fractures	Longitudinal, prospective	60 mg, follow up after 6 months (+ vitamin D)	/	Improved gait speed (0.06 m/s), TUG (1.7 s) and FSST (1.7 s); slight improvement in SPPB score (1.1 points)	[84]
78 men and 123 women with osteoporosis aged ≥ 65 years	Two-year, double-blind, placebo-controlled, randomized trial—PROUD trial	60 mg every 6 months for 2 years (+ Ca and vitamin D)	/	No statistically significant differences between the Denosumab and placebo groups in appendicular lean mass, chair stand performance, SPPB scores and gait speed	[86]

### 4.2. Bisphosphonates

Bisphosphonates are a family of potent inhibitors of bone calcification and bone resorption, and they are used orally and intravenously for the treatment of osteoporosis, glucocorticoid-induced osteoporosis and other skeletal disorders characterized by increased bone resorption (e.g., hypercalcemia, bone malignancies) [90]. Several formulations prescribed for osteoporosis treatment exist, including alendronate, risedronate, zoledronic acid and ibandronate, each with slightly different potency, dosing, regimens and indications. Bisphosphonates have a structure similar to that of pyrophosphate, a natural circulating inhibitor of mineralization, and can bind to the hydroxyapatite matrix on the bone mineral surface, from which they are released upon bone resorption. There, they suppress osteoclast activity by inhibiting farnesyl pyrophosphate synthase, which leads to the loss of function of osteoclasts, osteoclast apoptosis and the inhibition of bone resorption [90]. As such, bisphosphonates prevent bone loss, and an increase in BMD can be observed as early as three months after treatment initiation [91]. However, as bone resorption and bone formation are coupled, bisphosphonate treatment also reduces bone formation to a certain extent. Bisphosphonate treatment thus primarily aims at the preservation of, if not gradual improvement in, bone mass and bone microstructure, which nevertheless results in a reduced risk of vertebral and non-vertebral fractures as early as six months after initial administration [92,93,94].

Bisphosphonates also affect muscle tissue, although the mechanisms are not yet clear. While certain studies suggest a direct effect on muscle cell metabolism and cell signaling [95,96], others propose that the effect is mediated indirectly, through the inhibition of bone tissue resorption and consequent reduction in released resorption-related proinflammatory osteokines, such as transforming growth factor β (TGFβ) and IL-6 [97,98]. Nevertheless, several studies have demonstrated that bisphosphonates can prevent muscle wasting due to aging and in different pathological processes, such as osteoporosis, immobilization- or unloading-induced bone and muscle loss [96,99] (Table 2), burn injuries, cancer and chemotherapy [100]. Focusing only on the routine bisphosphonate treatment of osteoporosis, studies have shown significant positive effects on muscle mass following treatment with zoledronic acid for 3 years [101] and increased muscle strength and increased balance in patients treated with alendronate [102]. Alendronate–calcitriol therapy combined with regular endurance exercise increased handgrip strength and lowered IL-6 serum levels in osteopenic women after 6 months of treatment [103]. Similarly, 1-year treatment with alendronate helped maintain appendicular muscle mass compared to control osteoporotic patients, where muscle mass significantly decreased [104]. On the other hand, several studies have failed to demonstrate positive effects of bisphosphonates on muscle tissue. Three-year alendronate or zoledronic acid treatment failed to increase bone mass and handgrip strength [83], and smaller cross-sections of skeletal muscles were reported in long-term users of different bisphosphonate formulations compared to in non-treated patients [105]. Bisphosphonate administration also failed to improve physical function during rehabilitation [106].

Bisphosphonates are in general well tolerated and are considered the first line of therapy but can result in severe side effects, such as atypical fractures of the femur or osteonecrosis of the jaw [107]. In 2010, the FDA thus advised drug discontinuation after 3–5 years of use [108], but as bisphosphonates bind and accumulate in bone tissue, they tend to inhibit bone resorption for several years after discontinuation. Not enough studies have analyzed the mechanisms and effects of prolonged or discontinued bisphosphonate treatment on muscle tissue, but more importantly, so far, no clinical trial has addressed this. Moreover, studies also indicate possible negative effects of bisphosphonates on muscle regeneration capability upon simultaneous muscle and bone injury [109,110], which can be an important factor affecting the choice of osteosarcopenia treatment, especially following bone fracture. As different formulations of bisphosphonates are used with slightly different effects, a systematic analysis of their effects on muscle tissue would be of great help to better assess if any of the formulations would be more suitable to simultaneously address muscle wasting and, equally important, if any should be avoided in osteosarcopenic patients.

**Table 2:** Overview of studies addressing the effects of different bisphosphonate formulations on bone and muscle tissue. Abbreviations: BMD—bone mineral density; LBM—lean body mass; TUG test—Timed Up and Go test.

Drug	Cohort	Study	Regime	Effects on Bone	Effects on Muscle	Ref.
Alendronate	58 community-dwelling osteoporotic women ≥ 65 yo	Randomized, open-labelled, active-comparator	35 mg/week, for 24 weeks	Increased lumbar BMD (3.9%) and femoral BMD (1.9%); decreased bone turnover markers (compared to baseline)	Increased dynamic balance, increased knee extension force (19%) and power (15%) and increased gait speed (2.6%); no effect on TUG, grip strength or appendicular muscle mass index (compared to baseline)	[102]
	199 osteoporotic patients (233 control patients)	Retrospective, case-controlled	35 mg or 5 mg/week for 1 year	Retained bone mineral content (significantly decreased in control patients)	Increased skeletal muscle mass index (2.5-fold), appendicular skeletal muscle mass (2.5-fold), lower limb muscle mass (4.4-fold) and total fat mass	[104]
	17 osteoporotic postmenopausal women ≥ 63 yo	Open-label, randomized, controlled	35 mg/week for 6 months	/	No differences compared to baseline in alendronate-alone group in grip strength, back extensor strength, Iliopsoas muscle strength, static or dynamic postural balance or TUG test	[73]
	38 osteoporotic postmenopausal women	Double-blind, placebo-controlled, randomized	5 mg/day for 1 year	Increase in BMD in lumbar spine (3.5%) and femoral neck (1.3%) compared to placebo; no effect on radial bone mineral content	No effect on physical performance parameters, leg extensor power, dynamic balance and cardiorespiratory fitness (VO_2_max)	[111]
	62 community-dwelling osteoporotic patients ≥ 80 yo (61 control patients)	Randomized, controlled, non-blind	70 mg/week (+ Ca and alfacalcidol) for 18 months	Increased BMD in lumbar spine and femoral neck compared to baseline	No increase in muscle strength; decrease in TUG and gait speed	[112]
	36 postmenopausal women with osteosarcopenia	Longitudinal study	5 mg/day (+ calcitrol) for 6 months	Increased lumbar BMD (2.62%); no change in femur BMD	Improved handgrip strength (33.5%)	[103]
	136 older patients	Longitudinal, multicenter, controlled, prospective study	70 mg ALD/week for 5 years (+ Ca and vitamin D)	Improved spine and hip BMD; no significant change in falls risk	Improved TUG (0.8 s), 4 m walk test and gait speed (0.07 m/s), which persisted for up to 1 year after treatment discontinuation	[88]
Risedronate	91 osteopenic postmenopausal women (93 control)	Randomized, controlled	150 mg/every 4 weeks (+ Ca and vitamin D) for 1 year	Increase in spine (1.9%), hip (0.9%) and femoral neck (0.09%) BMD compared to baseline	Increased body fat; small increase in total LBM (control patients lost total LBM)	[113,114]
Zoledronic acid	62 older osteoporotic women ≥ 70 yo in long-term care communities	Double-blind, randomized, placebo-controlled	One 5 mg i.v. (+ Ca and vitamin D)	Increased spine (6%) and total hip (2.8%) BMD compared to baseline	No change in appendicular lean mass compared to control; slight decrease compared to baseline (−0.75%)	[115]
	136 older patients	Longitudinal, multicenter, controlled, prospective study	5 mg/year for 3 years (+ Ca and vitamin D)	Improved spine and hip BMD; no significant change in falls risk	Improved TUG (0.7 s), 4 m walk test and gait speed (0.07 m/s), which persisted for up to 1 year after treatment discontinuation	[88]
	1000 ambulant osteoporotic postmenopausal women > 65 yo	Double-blind, placebo-controlled	4 5 mg i.v. in 18-month intervals for 6 years	Reduced risk of fractures	Reduced weight loss, no change in fat mass and higher loss of LBM compared to placebo	[116,117]
	113 treated and 118 controls (both with osteoporosis)	Case–control retrospective cohort study	5 mg/year for 3 years	Significantly improved BMD	Significantly improved appendicular skeletal muscle mass and appendicular skeletal muscle index	[101]
	28 community-dwelling elderly patients (≥65 yo) with history or risk of falls and/or fractures	Longitudinal, prospective	5 mg i.v., follow up after 6 months (+ vitamin D)	/	Improved gait speed (0.1 m/s) and TUG (1.6 s)	[84]
Ibandronate	Children and adolescents (7–16 yo) with osteogenesis imperfecta	Longitudinal	3 mg/kg body weight i.v. every 4 months for 2–4 years	Increase in lumbar spine BMD and vertebral area and decreased fracture rate	Increased grip force, median mobility score and self-care score	[118,119,120]

### 4.3. Steroid Hormones and Hormone Replacement Therapies

One of the major causes of osteoporosis in elderly people is the decline in sex hormones [121]. In women, the hormones drop quickly upon menopause, while in men, the concentration of both testosterone and estrogens declines gradually, starting in their fifth decade [122]. As such, hormone replacements are being used to prevent hormone-deficiency-related symptoms and health problems, including bone loss both in men (hypogonadism) and in women (menopause) younger than 60 years for up to 10 years. Hormone replacement therapy (HRT) is able to preserve or even increase BMD at all skeletal sites and reduce the risk of fractures [123]. Unfortunately, hormone supplementation is also associated with the potential risk of long-term side effects such as breast or testicular cancer and an increased risk of cardiovascular and cerebrovascular events and is thus not advised for long-term therapy for the sole prevention of bone loss [124,125].

**Figure 2:**
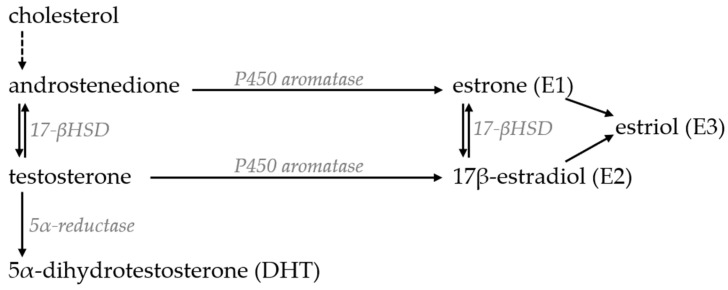
Schematic representation of steroid sex hormone synthesis pathway. All steroid hormones are synthetized from cholesterol, which is converted over a series of enzymatic reactions to dehydroepiandrosterone (DHEA) and further to androstenedione. Androstenedione is a direct precursor of both estrone and testosterone. Androstenedione is converted to testosterone and further to 5α-dihydrotestosterone (DHT), a biologically more active form of testosterone, through the action of hydroxysteroid 17-β dehydrogenase (17-βHSB) and 5α-reductase enzymes. Androstenedione and testosterone are also converted to estrone (E1) and 17β-estradiol (E2), respectively. Through reduction, estrone is then converted to estriol (E3).

The metabolism of these hormones is tightly connected, and different hormones can be converted through a series of reversible enzymatic reactions into any other biologically active form (Figure 2) [126]. As such, a reduction in the concentration of one hormone can affect the concentration of another and vice versa—the supplementation of a hormone can also enable an increase in other androgens or estrogens.

**Figure 3:**
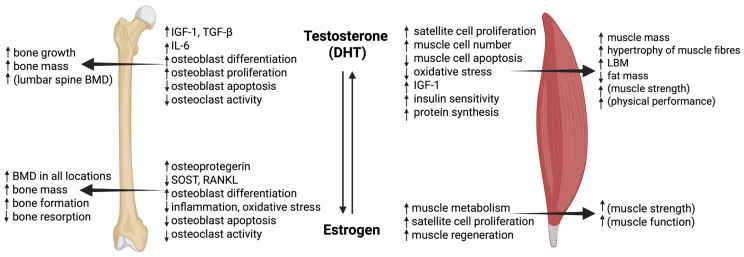
Schematic representation of the mechanisms and effects of testosterone and estrogens on bone and muscle tissues. The effects of testosterone are mediated directly or through its conversion to 5α-dihydrotestosterone (DHT) and binding to androgen receptors. The effects of estrogens are mediated through their binding to estrogen receptor-alpha (ERα) and -beta (ERβ), with ERα being dominant in the regulation of bone metabolism. Receptor binding results in changes in cell signaling, gene transcription and target-cell metabolism, factor secretion activity or differentiation. This can lead to measurable anatomical or physiological changes in the quality and function of bone or muscle tissue, as shown in the scheme. Abbreviations: IGF-1—insulin-like growth factor-1; TGFβ—transforming growth factor β; IL-6—interleukin 6; BMD—bone mineral density; SOST—sclerostin; RANKL—Nuclear factor-kappa B (NF-κB) receptor activator ligand; LBM—lean body mass. Arrows ↓ indicate decrease and ↑ indicate increase in the listed function or parameter, brackets ( ) indicate a frequently observed, but conflicting effect.

Although the concentrations of testosterone and estrogens are significantly different in men and women, both are required for normal bone growth and development (Figure 3). Estrogen is the dominant hormone regulating BMD and bone metabolism, while testosterone has been linked to bone growth, an increase in bone mass and periosteal apposition during adolescence, which results in larger bones in men than in women [127,128]. Accordingly, several studies have shown that estrogen deficiency is primarily responsible for the loss of BMD in aging patients of both genders. Serum estrogen levels decline significantly also in aging men and are more closely associated with BMD then testosterone levels. Men with mutations in the estrogen receptor Erα or aromatase enzyme or lacking estrogen can have osteoporosis despite having normal testosterone levels, which can be reversed with estrogen supplementation [129,130,131]. 5α-reductase and aromatase are widely expressed in the bone tissue, which indicates that weaker circulating forms of hormones can also be converted to biologically active metabolites directly in the bone microenvironment [132]. In this chapter, both hormones will be discussed separately; however, the reader should keep in mind that many of the positive effects of testosterone supplementation on BMD are most probably mediated by its conversion to estrogen.

#### 4.3.1. Testosterone

Testosterone replacement therapy (TRT) is used primarily in patients with diagnosed hypogonadism (low or borderline low testosterone < 280–300 ng/dL), which is a major cause of osteoporosis in men, and for the treatment of Hypoactive Sexual Desire Disorder/Dysfunction (HSDD) in women [133]. Studies on testosterone supplementation have shown conflicting results regarding BMD, and TRT is not currently an approved treatment for osteoporosis or fracture prevention in patients with normal testosterone concentrations, nor should it be prescribed as the sole treatment for osteoporosis in already-osteoporotic men regardless of their testosterone concentration [134]. Recently, four meta-analyses were performed analyzing the effects of testosterone on bone parameters, which showed inconclusive and conflicting results attributed to high heterogenicity in the studied populations, duration and treatment (testosterone application and dosing) [135,136,137,138]. In general, the greatest improvements in BMD were observed in men with the lowest serum testosterone concentrations (T < 264–300 ng/dL) [139,140], while the effectiveness of testosterone treatment against osteoporosis and fracture prevention in eugonadal men was less consistent. The highest increases in BMD were detected in the lumbar spine and to a lesser extent in the hip and femoral neck [136,140,141,142,143], which was attributed to a slower response in those areas. The increase in BMD following testosterone treatment also depends on the administered dose [141], but only to a certain degree, as the effect of testosterone is also determined by the number of available androgen receptors [144,145] and the activity of converting enzymes, such as 5α-reductase and aromatase [146,147] (Figure 2). It is thus not surprising that despite several promising studies, the effects of testosterone on BMD and fracture risk remain controversial.

In contrast to bone, testosterone has a strong anabolic effect on muscle tissue (Figure 3) [148,149]. In clinical trials, testosterone treatment has frequently been shown to induce an increase in lean body mass (LBM) and decrease in fat mass but, less consistently, also lead to increased strength and physical performance [150,151,152,153]. In a double-blind placebo-controlled trial of osteoporotic and frail men, testosterone gel application for 12–24 months increased BMD and LBM and decreased fat mass, but there were no differences in strength or physical performance [150]. In a similar 3-year study, only LBM increased, with no difference in the strength of knee extension and flexion between treated and control groups [139]. On the other hand, the oral administration of testosterone for 3 years not only increased LBM but also significantly improved muscle performance in timed functional tests and handgrip strength [154]. The effects of testosterone were more pronounced in men with lower baseline testosterone levels [153]. Testosterone supplementation was shown to also increase muscle mass and strength in secondary sarcopenia related to heart failure, chronic obstructive pulmonary disease, cancer, obesity, type 2 diabetes mellitus and other conditions [155].

In a similar way to bone, the response to testosterone in muscle is also dependent on the administered dose, route of administration and physical activity. The effects of testosterone are in general positively correlated with its dose both in terms of the size of the effect [156] as well as the muscle groups involved [141]. Skinner et al. (2018) showed that intramuscular administration resulted in a significantly higher increase in LBM and total body strength as well as upper- and lower-extremity strength compared to transdermal administration, which induced no increase in lower-extremity strength compared to the placebo [152], although a recent meta-analysis showed more consistent positive effects for transdermal and oral supplementation [149]. The third factor that affects especially the increase in physical performance is the physical activity of patients, both performed as an intervention alongside testosterone treatment as well as the baseline physical state of the patients, which greatly determines their ability to perform physical activity during the intervention [157]. Bhasin and co-workers noticed that older men with a higher baseline gait speed showed significantly greater improvement in gait speed compared to men with lower physical performance at the start of the study [158]. This was also confirmed in a placebo-controlled randomized study, which showed that testosterone supplementation in elderly men combined with an exercise program greatly improved their physical function, social functioning and mental and general health, while there were no significant effects of testosterone or exercise alone [159]. Testosterone thus acts synergistically with muscle physical activity and mechanical loading, but unfortunately, not many studies or clinical trials have determined physical activity at baseline and after the intervention.

One of the reasons for the limited use of testosterone and the skepticism regarding its potential use in the treatment of musculoskeletal disorders is its safety profile. In general, testosterone application is associated with a low frequency of serious adverse effects, such as erythrocytosis, an increase in prostate-specific antigen (PSA), prostate enlargement and an increased risk of prostate cancer, mainly due to its non-selective action [160]. However, the risks of serious adverse effects increase in patients with a high risk of prostate cancer, already-present prostate or breast cancer, palpable prostate nodules, already-high PSA and certain hearth issues—for these patients, TRT is not recommended [134]. Due to low effects, conflicting results of testosterone replacement therapies, possible side effects and the fact that available antiresorptive and osteoanabolic agents can effectively prevent BMD decrease due to testosterone decline, the Endocrine Society recommended the use of other approved therapies for low BMD for hypogonadal men unless they cannot be used due to contraindications or very low testosterone levels (<200 ng/dL) in patients [161].

#### 4.3.2. Estrogens

The sudden decrease in estrogen associated with menopause is one of the main triggers of age-related osteoporosis in women. Estrogen deficiency results in decreased osteoblast bone formation activity and increased osteoclast resorption activity, leading to a net loss of bone mass and BMD. HRT, either estrogen alone or in combination with progesterone, is thus an effective mechanism for the prevention of menopause symptoms, including osteoporosis, and considered as the first-line choice for many younger and early-menopausal women [162].

The beneficial effects of HRT in preventing menopausal symptoms have been confirmed in several successful clinical trials performed in the last 60 years. All trials showed a significant improvement in BMD compared to control patients at all measured sites, which increased with each year of treatment. In the Women’s Health Initiative (WHI) trial, the biggest randomized controlled clinical trial so far enrolling more than 10,000 postmenopausal women aged 50–79, the administered conjugated equine estrogen (CEE) 0.625 mg/day + medroxyprogesterone acetate (MPA) 2.5 mg/day increased hip BMD by 3.7%, lumbar spine BMD by 4.5% and total hip BMD by 3.6% after 3 years of treatment compared to the baseline, while BMD in the placebo group decreased [123]. Similar results were also obtained by recent meta-analyses including several smaller clinical trials in different settings [163,164]. Moreover, HRT also significantly reduced the risk of fractures [165]. For example, in the WHI trial, the hip fracture risk was lowered by 33% and the overall fracture risk by 24% during the 5-year observation period [123].

Similar to bone, estrogen receptors are also expressed in skeletal muscle satellite cells and differentiated myofibers, which suggests that estrogens can affect muscle tissue [166], but despite several studies in rodents and in humans, the mechanisms are still not completely understood. Muscle loss in women starts upon menopause onset, but the rate eventually slows down [167,168]. This is attributed to increased protein degradation [169] and reduced response to anabolic stimuli such as exercise and nutrition [170,171]. HRT is thus expected to affect muscle aging and potentially slow or prevent the emergence of sarcopenia, although not all human studies and clinical trials confirmed this effect. In general, HRT had beneficial effects on muscle strength in postmenopausal women that were strongly dependent on the muscle group [172], even though not all changes were statistically significant compared to the placebo [173]. The WHI trials showed positive effects of HRT in the shorter term (3 years), but no differences in LBM were detected between HRT and the control after 6 years of therapy [174,175]. Other studies showed the positive effect of HRT on muscle performance by improving vertical jump height, running speed and muscle strength [176,177]. On the other hand, when HRT was administered to older women (70–79 years old), it had only minor effects on muscle composition and strength and no effect on physical function [178]. This inconsistent response could be explained by different HRT concentrations, hormone combinations, the age of the patients and the time interval between menopause onset and the start of HRT. Moreover, baseline physical fitness and health, as well as additional supplements and exercise, might greatly influence the results, as studies have shown that physical training and HRT have synergistic effects compared to HRT or exercise alone [176,179]. HRT for muscle tissue preservation might thus be more efficient if started earlier, before the loss of muscle mass and strength occur, although it might not be able to prevent sarcopenia in the long term per se [174,180].

HRT is related to serious adverse effects including breast cancer, coronary heart disease, strokes and thromboembolisms [181,182], which however can be significantly reduced if HRT is administered in younger women (less than 60 years old) and shortly after the onset of menopause, when the bone loss is the fastest [183]. Still, to prevent osteoporosis, the lowest effective dose should be used, and patients should be regularly assessed to weigh the changing benefits and risks of the treatment. Unfortunately, the withdrawal of therapy results in rapid bone loss within the following years, which can exceed the gains obtained during active HRT [184,185], so other antiresorptive therapies should be considered to retain the gains in BMD for as long as possible.

#### 4.3.3. Selective Estrogen Receptor Modulators

Selective estrogen receptor modulators (SERMs) are synthetic non-steroidal molecules that can act as both estrogen receptor agonists and antagonists, depending on their structure and target tissues. This selectivity gives SERMs an advantage over estrogen therapy, as they affect breast and endometrial tissue to a lesser extent [186]. Several SERM formulations have been developed and FDA-approved so far, but based on their tissue-specific activity, only raloxifene and bazedoxifene have been prescribed for the treatment of osteoporosis. Tamoxifen can also prevent bone loss in postmenopausal women [187], but due to its severe side effects, it is only approved for the prevention and treatment of breast cancer [188]. Similarly, Lasofoxifene showed positive effects on BMD and reduced vertebral and non-vertebral fractures [189,190] but was rejected by the FDA due to safety concerns and insufficient data in 2009 [191].

Raloxifene was the first SERM approved for the treatment and prevention of osteoporosis in postmenopausal women. It acts as a partial estrogen receptor agonist in bone, preventing bone loss and reducing vertebral fractures [192,193]. In the randomized, blinded and placebo-controlled clinical trial MORE, 3-year treatment with raloxifene maintained BMD and minimized bone loss in the spine and femoral neck of postmenopausal women, which resulted in a 30% reduction in vertebral fractures but had no effect on the rate of hip fractures [192,194]. The risk of vertebral fractures was reduced already after 3 months of treatment [195]. Continued raloxifene therapy maintained the initial gains in BMD in the spine and hip but showed minimal further improvements, as demonstrated by the Continuing Outcomes Relevant to Evista (CORE) study [196]. The increase in BMD and bone turnover following raloxifene treatment is generally lower compared to HRT [197,198], and it only appears to decrease fracture incidence in the spine. The discontinuation of treatment results in bone loss comparable to or greater than that in non-treated women [199,200].

Bazedoxifene (BZD) is a third-generation SERM that has been shown to increase BMD in the spine, total hip, femoral neck and greater trochanter [201,202,203,204], reduce bone turnover markers [201,205] and reduce the risk of vertebral fractures [202,203,204,206]. It was FDA-approved for prevention in 2007 and for the treatment of postmenopausal osteoporosis in 2008, and since 2013, BZD has also been approved as a part of a combination drug of 0.45 mg conjugated estrogens (CEs) and 20 mg BZD per tablet [207]. This combination increased the tissue-selective activity of each component and reduced the side effects, with additional positive effects on menopausal symptoms while avoiding the use of progestin. This approval was based on five extensive phase III Selective Estrogen Menopause and Response to Therapy (SMART) clinical trials, in which this complex showed the prevention of bone loss (increased lumbar spine, femoral neck and total hip BMD), a reduction in bone turnover markers, a reduction in vertebral fractures and no increase in endometrial side effects regardless of the dose used. The positive effects of BZD/CE were seen already after 6 months, and efficacy was retained for the whole duration of the studies with minimal side effects. More positive effects were observed in high-risk women with already-present vertebral fractures and low BMD [208,209,210,211,212]. Unfortunately, due to its effects being limited to vertebral fractures and relatively low gains compared to other antiresorptive drugs, BDZ is usually not considered as the first treatment choice for most postmenopausal women [213].

Despite promising results obtained with osteoporosis treatment, minimal research has been published on the effects of SERM on muscle tissue (Table 3). One year of randomized, controlled raloxifene treatment caused changes in the body composition of healthy postmenopausal women, increasing the fat-free mass and total body water, but did not significantly change the BMI, muscle strength or power [214]. Similarly, in another randomized, double-blind, placebo-controlled clinical trial involving elderly women, raloxifene did not show an effect on handgrip muscle strength [215]. In another small, prospective randomized control trial in postmenopausal women, raloxifene retained the total fat mass (while it increased in the control group) but had no effect on total, arm or leg LBM [216]. To the best of our knowledge, BZD was not evaluated for its effects on muscle tissue. SERMs do not seem to induce noticeable improvements in muscle mass, strength or function, which would have prompted further research in this area.

The development of SERMs with estrogen-like activity restricted to bone tissue is still under way. The present formulations have shown promising results on bone, although the compounds can still have serious adverse side effects, such as thromboembolic events and uterine and breast cancer. Moreover, the present formulations do not seem to be active in human muscle tissue, as no effects were detected on muscle mass or muscle strength in the few studies published so far. This could be due to the binding and tissue specificity of the current SERM formulations or the fact that SERMs cannot be converted to testosterone like estrogens. On the other hand, several selective androgen receptor modulators (SARMs) also exist with positive effects on muscle tissue, but none are FDA-approved due to inconsistent action and safety concerns [217]. Due to the general positive action of androgens on muscle tissue compared to estrogens, novel and safer SARM formulations might be a better way to address sarcopenia [218].

### 4.4. Teriparatide and Abaloparatide

Teriparatide (PTH), a recombinant human parathyroid hormone (1–34), and abaloparatide (ABL), the parathyroid-hormone-related protein analogue, are FDA-approved bone anabolic drugs for the treatment of postmenopausal women, men with primary or hypogonadal osteoporosis and patients with glucocorticoid-induced osteoporosis who are at high risk of fractures [219,220]. Both are activators of the PTH type 1 receptor signaling pathway and have similar anabolic effects on bone tissue [221]. The intermittent application of PTH or ABL stimulates both bone formation and bone resorption, but bone formation outweighs bone resorption, which results in a net increase in bone mass and bone strength. PTH and ABL also affect calcium and phosphate homeostasis through their effects on bone and kidneys and are thus co-administered with calcium and vitamin D supplements. Due to several similarities, only PTH as the older drug with more clinical trials will be discussed herein.

Several preclinical and clinical trials have shown positive effects of PTH treatments on bone tissue both in older men and postmenopausal women [222,223]. PTH mostly induces an increase in trabecular bone BMD, which is reflected by a greater BMD increase in the spine than in the hip [224]. In a seminal multicentered, randomized, placebo-controlled trial in postmenopausal women (*n* = 1637), 20 μg/day PTH increased lumbar spine BMD by 9% and femoral neck BMD by 3% during the 21-month study duration. This resulted in a reduced risk of one or more vertebral fractures by 65% at a 20 μg/day dose and by 69% for a 40 μg/day dose and by 35% and 40% for non-vertebral fractures, respectively [222]. Several following randomized and observational studies confirmed these effects [225] and showed that PTH was even more effective in increasing BMD than bisphosphonates or their combinations [226]. Nevertheless, bisphosphonate antiresorptive treatment is usually started after the cessation of PTH, which helps retain the BMD gained for longer [227,228].

PTH also affects muscle tissue. It increases muscle protein catabolism, impairs energy production by reducing oxygen uptake in mitochondria and increases free calcium in the muscle tissue [229,230]. Despite this, only a few human studies and clinical trials have also looked at the effect of PTH treatment on muscle, mostly as a secondary outcome (Table 4). In a phase IV, randomized, multicenter, active-controlled trial comparing the effects of PTH and risedronate on pertrochanteric hip fracture recovery also showed that the time required to complete the TUG test was significantly shorter with PTH treatment compared to with risedronate at all assessed time points with significantly lower reported hip pain [231,232]. Similar results were also obtained in elderly osteoporotic women with pubic bone fractures treated with PTH compared to an untreated cohort [233] and in a small cohort of patients with a low-trauma femoral neck fracture repaired with internal fixation, where a greater proportion of patients treated with PTH were able to retain or regain their ability to walk [234]. On the other hand, a study investigating the effects of whole-body vibration exercises and PTH treatment in postmenopausal women with severe osteoporosis only showed improvement in the SPPB (short physical performance battery) in the combined treatment group and improved leg extension power in the combined and PTH only groups but no change in the TUG test, grip strength or LBM in either group compared to the baseline [235]. Similarly, no improvement compared to the placebo cohort was observed in pain scores, functional tests or grip strength in postmenopausal women with a distal radius fracture [236].

Although PTH affects muscle metabolism and signaling at a cellular level, clinical studies mostly indicate that PTH treatment is associated with better and early functional outcomes but does not affect muscle mass, function or structure in terms of sarcopenia prevention or treatment. Not enough studies have been performed to understand the effects of PTH on muscle tissue and function (and none for ABL), but in either case, significant effects of PTH treatment on sarcopenia-associated muscle properties most probably cannot be expected.

### 4.5. Romosozumab

Romosozumab (formerly AMG 785/CDP7851) is a humanized IgG2 monoclonal antibody against sclerostin (SOST), an endogenous inhibitor of the Wnt signaling pathway. By binding to SOST, romosozumab prevents its inhibitory function and thus increases Wnt pathway signaling activity, leading to increased bone formation [238]. Romosozumab is thus prescribed to patients with very low BMD and a very high risk of fractures, where just retaining the current BMD with antiresorptive treatment would not suffice. In such patients, bone quality is first increased with 1-year romosozumab treatment, which is followed by a potent antiresorptive therapy (Denosumab, alendronate, zoledronic acid) to retain the increase in BMD and lowered fracture risk for longer [239,240].

Several clinical studies have demonstrated a quick and significant increase in BMD in both healthy and osteoporotic men and women. For example, in a phase 2 clinical trial, the BMD increased by 16.9% and 4.7% in the lumbar spine and total hip, respectively, after 1 year of treatment with monthly 210 mg romosozumab injections in osteoporotic postmenopausal women [241]. Romosozumab also positively affected bone structure as measured by high-resolution quantitative computed tomography (HR-QCT) in a phase 1b placebo-controlled clinical trial, where vertebral trabecular BMD and stiffness increased by 9.5% and 26.9% from the baseline, respectively, already after 3 months [242]. The increased bone mass and bone metabolism led to a significantly reduced fracture risk for both vertebral and non-vertebral fractures compared to the placebo [243]. In parallel comparison clinical studies, romosozumab performed better than PTH, Denosumab or alendronate, making it the most effective anabolic drug available for the treatment of osteoporosis [244,245,246].

Although primarily secreted from osteocytes in bone tissue, SOST is also expressed by and can affect muscle cells [247]. In muscle, the Wnt signaling pathway regulates muscle development, repair and homeostasis during adult life [248] but also affects adipose tissue [249]. As romosozumab was only FDA-approved in 2019, none of the clinical studies performed so far have also addressed its effects on muscle tissue, and the current knowledge of SOST dynamics in the regulation of muscle metabolism is inconsistent. In an observational cross-sectional study of a community-dwelling elderly Korean population, the serum SOST levels were significantly lower in participants with sarcopenia, low muscle mass and strength and were positively associated with the skeletal muscle index and grip strength [250], suggesting an opposite metabolic effect of SOST on muscle compared to bone. In contrast, two similar studies of sarcopenic obese non-diabetic and diabetic adults showed that low muscle mass was associated with higher SOST levels [251,252]. Moreover, a recent meta-analysis indicated that romosozumab can reduce the risk of falls in postmenopausal women [253], suggesting that romosozumab could positively affect muscle function, but on the other hand, it did not affect recovery or physical performance in the TUG test following hip fracture [254].

Romosozumab is a highly efficient bone anabolic drug with minimal side effects for the treatment of osteoporosis, but not enough is known regarding its effects on muscle tissue. As studies have confirmed that muscle tissue can also secrete and respond to SOST at a cellular level, future studies should not only look at its effects on muscle mass, quality and strength but also address muscle adiposity, turnover markers and the possible additive effects of physical activity and loading. Despite this, major positive effects of romosozumab on muscle tissue are not expected.

## 5. Myostatin Inhibitors

Although this review focuses on drugs for osteoporosis, recently, the first drug targeting muscle tissue wasting directly has been approved, and this prompted us to add this chapter. Taldefgrobep alfa is a muscle-targeted recombinant protein which acts as an inhibitor of both myostatin and activin A signaling, two key regulators of muscle and adipose tissue growth and development. Taldefgrobep alfa can lead to a reduction in fat mass, increased LBM and improvements in multiple metabolic parameters. In 2023, taldefgrobep alfa was granted a Fast Track and Orphan Drug Designation by the FDA as well as an EU Orphan Drug Designation for the treatment of spinal muscle atrophy (SMA), and the phase 3 clinical trial RESILIENT is currently under way to test the efficacy and safety of this drug as an adjunctive therapy to increase muscle mass in SMA patients (CinicalTrials.gov Identifier: NCT05337553) [255,256].

**Figure 4:**
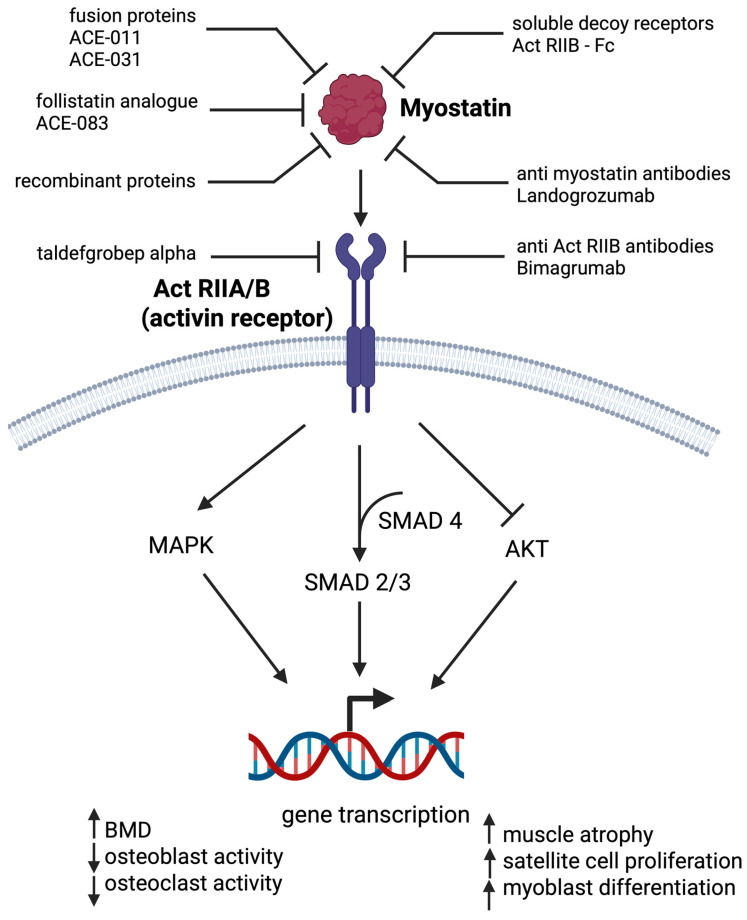
Schematic representation of myostatin signaling and mechanisms of its repression. Myostatin is a soluble myokine that acts as a negative regulator of muscle growth and mass by binding to activin type II receptors (ActRII A/B) on the cell. This binding activates a signaling cascade involving several different kinases such as Mitogen-activated protein kinase (MAPK), Sma- and Mad-Against Decapentaplegic (SMAD)-related proteins and protein kinase B (AKT). The activated proteins then translocate to the nucleus where they affect target gene expression, resulting in negative changes in muscle and bone tissues. Blocking myostatin binding to the receptor through different pharmaceutical approaches results in increased muscle and possibly also bone quality.

The targeted protein myostatin is a muscle-secreted cytokine that acts as a master regulator of skeletal muscle mass but also affects muscle fiber type composition and satellite cell proliferation. Studies have shown that myostatin affects myofibers directly by binding to the two activin type 2 receptors (2A and 2B) (Figure 4) and limits myofiber growth, while the inhibition of myostatin or its receptors enables myofiber and muscle growth without the activation of satellite cells [257]. A loss-of-function mutation in the myostatin gene has been associated with muscle hypertrophy in humans [258], and several animal studies have confirmed that the knock-out of the gene improved muscle mass and in certain cases also muscle function [259,260]. This suggests that myostatin inhibition could represent a potential mechanism to counteract age-related sarcopenic muscle loss.

Many myostatin-inhibiting agents have been developed, and some have also been evaluated in clinical trials (Figure 4). Unfortunately, although many of them induced the desired increase in muscle mass, these improvements were modest compared to the effects of the same drugs seen in animal studies, and most of them also failed to show significant functional improvements [260]. In a phase 1 study on healthy subjects and pediatric patients with neuromuscular disease, taldefgrobep alfa treatment increased thigh muscle volume and total LBM and showed a favorable safety profile [261,262]. A lot of trials on LBM improvement were performed also with bimagrumab (BYM338), a human monoclonal antibody, which binds and inhibits activin receptor 2B. Two small clinical trials showed that bimagrumab treatment in community-dwelling elderly people with sarcopenia increased muscle mass and strength and improved mobility in those with a slower walking speed at baseline compared to the placebo [263] but induced no additional substantial improvement if combined with adequate nutrition and light exercise [264]. Similarly, bimagrumab treatment led to a significant increase in LBM but with no positive effect on functional capacity in older patients recovering from hip fracture surgery [265] or elderly obese patients [266]. Despite this, its development for the treatment of sarcopenia was discontinued in 2018 due to safety concerns. Next, Landogrozumab (LY2495655), a humanized monoclonal antibody targeting myostatin, induced a significant increase in LBM and decrease in fat mass in patients undergoing elective total hip arthroplasty [267]. Improvements in LBM and physical performance were seen also in elderly patients who had fallen in the past [268]. Demagrozumab, another monoclonal anti-myostatin antibody, was developed and tested in phase 2 clinical trial in boys with Duchenne muscular dystrophy, but as it only resulted in non-significantly increased muscle volume and no improvement in muscle function, the trial was terminated early [269]. ACE-083, a locally acting follistatin-based myostatin inhibitor, significantly increased the volume of the muscles into which it was injected but did not affect muscle strength in postmenopausal women [270] or in patients with facioscapulohumeral muscular dystrophy [271] and was thus discontinued.

Despite primarily being a muscle-secreted and -acting protein, myostatin and its activin receptor have also been shown to affect bone tissue [272,273]. In animal studies, myostatin knock-out in aging mice resulted in increased BMD, bone mineral content and bone area as compared to wild-type controls [274]. Decoy forms of activin receptors or follistatin-based molecules that block myostatin and activin signaling have been shown to cause a rapid and significant increase in bone density, which has been attributed to the decreased inhibition of osteoblasts and increased inhibition of osteoclasts [16,275]. For example, a soluble myostatin decoy receptor (ActRIIB-Fc) increased bone mass in wild-type and osteogenesis imperfecta mouse models [276] and in normal and ovariectomized mice [277,278]. In humans, only two clinical studies have also looked at the effects of myostatin inhibition on bone tissue. Sotatercept (ACE-011), a fusion protein of activin receptor type IIB and IgG1-Fc, increased bone-specific ALP and reduced bone resorption biomarkers in postmenopausal women in a randomized, double-blind, placebo-controlled study [279]. Similarly, in boys with Duchenne muscular dystrophy, a trend in increased spine BMD was observed with an increasing concentration of ACE-031, a fusion protein of activin receptor type IIB and IgG1-Fc, which binds myostatin [280]. Unfortunately, the development of both formulations was stopped. Based on the reported results of the animal studies and the few formulations tested in patients, more attention should also be given to the possible effect of myostatin inhibitors on the bone tissue, as a possible treatment option, as well as unwanted side effects.

One of the limitations of myostatin inhibitors is the inhibiting activity on other similar members of the TGF-β family, such as bone morphogenic protein 9 (BMP9), BMP10 [280] or BMP11 [281]. This can result in side effects and damage to other tissues, including endothelial dysfunction [280] and loss of BMD [281]. Further research is thus needed to improve myostatin specificity, which may provide better therapeutic benefits with enhanced safety profiles. Nevertheless, although there are currently no approved myostatin inhibitors for the treatment of sarcopenia due to still limited efficacy, especially in terms of increasing physical performance, several studies and clinical trials have demonstrated that the inhibition of the myostatin and activin signaling pathways is still a promising area for further research into novel drug formulations for sarcopenia and possibly also osteosarcopenia.

## 6. Conclusions

Several in vitro molecular studies have shown that drugs for the treatment of osteoporosis can affect muscle tissue at least at molecular and cellular levels. In some cases, these effects are also translated at a physiological level through measurable changes in muscle structure and function. However, these effects are complex and dependent on several treatment and patient parameters which we do not fully understand yet. Expectedly, not many clinical studies have addressed the effects of osteoporosis drugs on muscle, and the performed studies frequently show high variability in both the study parameters and obtained results, which makes it difficult to draw reliable conclusions at this stage. Nevertheless, studies show potential positive effects of certain drugs discussed in this review, which are summarized in Table 5. The most promising results on muscle tissue were obtained with Denosumab and HRT, while the effects of bisphosphonates are less clear and would require a more systematic approach to address the effects of each bisphosphonate formulation separately. Only minor if any effects on muscle were observed following teriparatide and romosozumab treatment. Regardless of the drug used, a lot of studies also emphasized the importance of not only the adequate supplementation of nutrients (vitamin D, calcium, proteins) but more importantly physical activity, which was shown to considerably improve muscle-related parameters but was rarely included as an intervention in the studies and routine treatment.

The approach of taking sarcopenia into account when choosing between equal options for osteoporosis treatment would also reduce the problem of polypharmacy in the elderly population—prescribing a drug that can address both bone and muscle tissues could reduce the burden on the aging body and possible interferences between other drugs taken simultaneously. This should also be considered for the future development of drugs for osteoporosis and sarcopenia, as the two diseases frequently occur simultaneously. Finding a drug that can address both bone and muscle tissues (and maybe directly or indirectly other tissues affected by aging) would thus not only lessen the burden on the patients but also decrease treatment costs and side effects.

## Figures and Tables

**Table 3:** Overview of studies addressing the effects of raloxifene on bone and muscle tissue. Abbreviations: BMD—bone mineral density; BMI—body mass index; LBM—lean body mass.

Cohort	Study	Regime	Effects on Bone	Effects on Muscle	Ref.
198 women aged 70–80 years	Randomized, double-blind, placebo-controlled	60 mg/day for 12 months	/	Increase in fat-free mass and total body water; no differences in BMI and fat mass. No significant differences in muscle strength or muscle power.	[214]
198 community-dwelling women aged > 70	Randomized, double-blind, placebo-controlled	60 mg/day for 24 months	Increased hip (0.011 g/cm^3^) and lumbar spine (0.02 g/cm^3^) BMD compared to placebo	No effect on handgrip muscle strength, muscle power, mobility measures and body composition compared to placebo.	[215]
45 postmenopausal women	Prospective, randomized, controlled	60 mg/day for 12 months	/	No changes in LBM; retained baseline fat mass (increased in control group).	[216]

**Table 4:** Overview of studies addressing the effects of teriparatide on bone and muscle tissue. Abbreviations: BMD—bone mineral density; LBM—lean body mass; VAS—visual analog scale; WBV—whole-body vibration exercise; SPPB—short physical performance battery; 5TSTS—5 times sit-to-stand test.

Cohort	Study	Regime	Effects on Bone	Effects on Muscle	Ref.
86 osteoporotic patients with hip fracture aged ≥ 50 years (+85 patients on risendronate)	Phase IV, randomized, multicenter, active-controlled trial	20 μg/day subcutaneous (+ calcium and vitamin D) for 6 months	Similar hip fracture healing rate compared to risendronate group (radiographic evidence)	Shorter time to complete TUG test and reduced hip pain compared to risendronate group	[231]
389 osteoporotic patients with pertrochanteric fracture (+85 patients on risendronate)	Multinational, multicenter, prospective, randomized, active-controlled	20 μg/day subcutaneous (+ calcium and vitamin D) for 1.5 years	Increased lumbar BMD compared to risendronate group (mean difference, 0.040 g/cm^2^), increased femoral neck BMD compared to baseline and no change in total hip BMD from baseline or risendronate group	Shorter time to complete TUG test and reported pain with VAS score at early time points; no difference compared to risendronate group when the fracture healed	[232]
21 patients with pelvic fracture (44 controls)	Prospective, randomized, controlled	100 μg of PTH 1–84/day (+ calcium and vitamin D)	Faster fracture healing compared to control (radiographic evidence)	Shorter time to complete TUG test and improved VAS score for pain compared to control group	[233]
78 patients with femoral neck fracture aged ≥ 50 years (81 control group)	Prospective, randomized, double-blind, placebo-controlled phase III	20 μg/day subcutaneous (+ calcium and vitamin D) for 6 months	No differences in radiographic healing between the teriparatide and placebo groups at 10 weeks, 6 months or 12 months	No statistical difference in pain scores, gait speed time or recovery to pre-fracture ambulatory status but with a trend of better recovery in teriparatide group	[234]
35 osteoporotic postmenopausal women aged ≥ 50 years with history of fracture	Randomized, controlled trial	20 μg/day subcutaneous alone or in combination with WBV for 1 year	Significant increase in lumbar spine BMD in teriparatide group (6.65% ± 5.51) and teriparatide + WBV (8.90% ± 5.47), increase in bone turnover markers in both groups and no change bone microarchitecture parameters	Improved SPPB, 5TSTS, leg extension power and time to walk three meters in teriparatide + WBV compared to baseline; teriparatide-only group showed an increase in leg extension power compared to baseline; no significant change in handgrip strength, TUG or total or appendicular LBM	[235,237]
102 postmenopausal women with distal radius fracture	Multinational, multicenter, prospective, randomized, controlled, double-blind study	20 or 40 µg or placebo/day for 8 weeks (+ calcium and vitamin D)	Radiographic evidence of healing detected at 9.1, 7.4 and 8.8 weeks in the placebo, teriparatide 20 µg and teriparatide 40 µg groups, respectively (not statistically different); no differences in radiologic and anatomic deformities	Significant improvement in pain scores, grip strength and functional test (patient-rated wrist evaluation score) in both treatment groups but no significant differences compared to placebo	[236]

**Table 5:** Summary of the effects of FDA-approved drugs for the treatment of osteoporosis on bone and muscle tissues. Abbreviations: BMD—bone mineral density; LBM—lean body mass; TUG—Timed Up and Go; FSST—Four-Square Step Test; SERM—selective estrogen receptor modulator.

Drug Type	Effects on Bone	Effects on Muscle
Denosumab	Rapid increase in femoral neck, lumbar spine, hip, trochanter and total body BMD through inhibition of osteoclast activity and reduction in vertebral and non-vertebral fractures	Indications of increased muscle mass, strength (improved handgrip strength) and function (gait speed, TUG, FSST)
Bisphosphonates	Prevention of further bone loss and potential gradual increase in BMD through suppression of osteoclast activity	Conflicting evidence
Testosterone	Suggested lumbar spine BMD improvement in men with low serum testosterone concentrations	Increase in LBM and decrease in fat mass; indicated increase in strength and physical performance if combined with physical exercise
Estrogen	Increase in hip, lumbar spine and total body BMD and reduced risk of fractures	Indicated preservation of or increase in LBM and muscle performance in younger women if treated immediately upon menopause onset
SERMs	Prevention of or increase in BMD loss in spine and femoral neck and reduction in vertebral fractures	Maintenance of or reduction in fat mass; no indicated effect on LBM, muscle strength or performance
Teriparatide, Abaloparatide	Increase in cancellous bone, reduced risk of vertebral and non-vertebral fractures and improved fracture healing	Better early functional outcomes following fracture healing
Romosozumab	SOST inhibition leading to effective increase in bone formation and decrease in bone resorption	No evidence

## Data Availability

Data sharing is not applicable.

## Abbreviations

The following abbreviations are used in this manuscript:

25(OH)D	25-Hydroxy-vitamin D (calciferol)
ABL	Abaloparatide
BMD	Bone mineral density
BMI	Body mass index
BZD	Bazedoxifene (SERM drug)
DHEA	Dehydroepiandrosterone
EMA	European Medicines Agency
ERα/β	Estrogen receptor-alpha/beta
FDA	Food and Drug Administration
FGF	Basic fibroblast growth factor
FSST	Four-Square Step Test
HRT	Hormone replacement therapy
LBM	Lean body mass
IL-6	Interleukin 6
IGF-1	Insulin-like growth factor-1
MSCs	Mesenchymal stem cells
NF-κB	Nuclear factor-kappa B transcription factor
OPG	Osteoprotegerin
PTH	Teriparatide
RANKL	Nuclear factor-kappa B (NF-κB) receptor activator ligand
SERMs	Selective estrogen receptor modulators
SMA	Spinal muscle atrophy
SOST	Sclerostin
SPPB	Short physical performance battery
TUG	Timed Up and Go muscle performance test
